# New CHA_2_DS_2_-VASc-HSF score predicts the no-reflow phenomenon after primary percutaneous coronary intervention in patients with ST-segment elevation myocardial infarction

**DOI:** 10.1186/s12872-020-01623-w

**Published:** 2020-07-25

**Authors:** Qin-Yao Zhang, Shu-Mei Ma, Jia-Ying Sun

**Affiliations:** Department of Cardiology, Shengjing Hospital of China Medical University, No.36, Sanhao Street, Heping District, Shenyang, 110004 Liaoning China

**Keywords:** CHA_2_DS_2_-VASc-HSF score, ST-segment elevation myocardial infarction, Primary percutaneous coronary intervention, No-reflow phenomenon

## Abstract

**Background:**

The no-reflow phenomenon (NRP) is a serious complication of primary percutaneous coronary intervention (PPCI) and is an independent predictor of poor prognosis. We aimed to find a simple but effective risk stratification method for the prediction of NRP.

**Methods:**

This retrospective single-center study included 454 consecutive patients diagnosed with acute ST-segment elevation myocardial infarction (STEMI) and treated by PPCI, who were admitted to our emergency department between January 2017 and March 2019. The patients were divided according to the post-PPCI thrombolysis in the myocardial infarction flow rate: the NRP group and the control group. The CHADS_2_, CHA_2_DS_2_-VASc, and CHA_2_DS_2_-VASc-HSF scores were calculated for all the patients in this study, and multivariable regression and receiver operating characteristic curve analyses were conducted to determine the independent predictors of NRP and the predictive value of the three scores.

**Results:**

A total of 454 patients were analyzed in this study: 80 in the no-reflow group and 374 in the control group. The incidence of NRP was 17.6%. Creatine kinase-myocardial band, Killip class, stent length, and multivessel disease also independently predicted NRP. The CHA_2_DS_2_-VASc-HSF score had a higher predictive value than the other two scores, and a CHA_2_DS_2_-VASc-HSF score of ≥4 predicted NRP with a sensitivity of 72.5% and specificity of 66.5% (area under the curve: 0.755, 95% confidence interval [0.702–0.808]).

**Conclusion:**

Although the CHADS_2_, CHA_2_DS_2_-VASc, and CHA_2_DS_2_-VASc-HSF scores can all be used as simple tools to predict NRP, our findings show that the CHA_2_DS_2_-VASc-HSF score had the highest predictive value. Thus, the CHA_2_DS_2_-VASc-HSF score may be an optimal tool for predicting high-risk patients.

## Background

ST-segment elevation myocardial infarction (STEMI) is a common critical illness associated with coronary artery disease, for which primary percutaneous coronary intervention (PPCI) has become the preferred treatment strategy [1]. In some cases, despite adequate reopening of the culprit lesion, the ischemic myocardium still cannot obtain effective perfusion. This is known as the no-reflow phenomenon (NRP), which renders PPCI less effective in patients with STEMI and is regarded as an independent predictor of short- and long-term morbidity and mortality [2, 3]. Due to the lack of an established risk evaluation method, NRP occurs in up to 60% of patients with STEMI [4, 5]. As a result, it is important to find a simple and effective risk stratification method for the prediction of NRP.

The CHA_2_DS_2_-VASc score (congestive heart failure, hypertension, age [≥65 = 1 point, ≥75 = 2 points], diabetes, stroke/transient ischemic attack [TIA] [2 points], and vascular disease [peripheral arterial disease, previous myocardial infarction [MI] and aortic atheroma]) was developed from the CHADS_2_ score and has been used extensively to predict thromboembolic events in patients with nonvalvular atrial fibrillation and determine whether to use anticoagulant or antiplatelet drugs [6]. The components of these two scores are associated with atherosclerosis, vascular spasm, and microvascular dysfunction, which overlap the risk factors of NRP [7]. Several recent studies have also shown that the CHA_2_DS_2_-VASc scores are associated with the severity of coronary artery disease, its prognosis, and NRP in patients with acute MI, contrast-induced nephropathy after PPCI for acute coronary syndrome, in-stent restenosis, acute stent thrombosis, mechanical mitral valve thrombosis, and right ventricular dysfunction [8–16].

Recently, a new score, known as the CHA_2_DS_2_-VASc-HSF score, was developed, which includes three new variables in addition to those comprising the CHA_2_DS_2_-VASc score: hyperlipidemia (H), smoking (S), and a family history of coronary heart disease (F). The female sex, as used in the previous scores, was also replaced by the male sex [17]. In the present study, we investigated whether the CHADS_2_, CHA_2_DS_2_-VASc, and CHA_2_DS_2_-VASc-HSF scores could predict NRP in patients with STEMI treated by PPCI and subsequently compared the accuracy of the three scores in predicting NRP.

## Methods

### Study population

This retrospective single-center study included 454 patients diagnosed with acute STEMI, treated by PPCI, and admitted to our emergency department between January 2017 and March 2019. The patients were divided into two groups according to the thrombolysis in the myocardial infarction (TIMI) flow grade after PPCI: The normal/slow-flow group comprised patients with a TIMI flow grade of > 2, and the no-reflow group comprised patients with a TIMI flow grade of ≤2, despite a successful reopening of the culprit vessel and a lack of mechanical obstructions that would reduce the coronary blood flow, such as thromboembolism, dissection, or spasm.

The diagnostic criteria for acute STEMI were as follows: (1) patients with typical angina symptoms lasting for more than 30 min and not totally relieved by nitroglycerin; (2) patients with a new ST-segment elevation in at least two contiguous leads, with elevation defined as ≥0.2 mV in men or ≥ 0.15 mV in women for leads V2 to V3 or ≥ 1 mm (0.1 mV) in the other leads or a new left bundle branch block; (3) patients with myocardial injury marker levels, including creatine kinase-myocardial band (CK-MB) or troponin higher than the normal upper limit within 12 h of the onset of symptoms; and (4) patients with symptoms that lasted less than 12 h [18]. The exclusion criteria were as follows: (1) patients who received thrombolytic therapy before PPCI; (2) patients in whom stent implantation was rejected; (3) patients with percutaneous coronary angioplasty only; and (4) patients with severe liver or kidney dysfunction. Patient data, including demographics and clinical characteristics, angiographic features, and laboratory findings, were obtained from the hospital records. This study was approved by our ethics committee and was given the ethical number 2019ps602k, with the number of people at 460. The TIMI flow grades are classically defined as follows: grade 0, no antegrade blood flow through the vascular occlusion; grade 1, a small amount of contrast agent can pass through the stenosis but cannot fill the distal coronary bed; grade 2, the contrast agent can fill the distal coronary bed, but the filling speed is slow; and grade 3, the contrast agent fills the distal coronary bed quickly and completely [19]. The TIMI flow grade was evaluated by two cardiologists who were blinded to the study. The frame rate of the cine images was 30 frames/s. The Killip grades are Class I patients who did not show evidence of heart failure (HF). Class II patients presented mild to moderate heart failure, but wet snoring occurred in less than 50% of the two lung fields. Class III patients exhibited severe heart failure, the presence of wet sputum was greater than 50% of both lungs, and acute pulmonary edema was a possibility. Class IV consisted of patients with cardiogenic shock [20].

All patients received 300 mg of aspirin and 600 mg of clopidogrel at the time of the diagnosis of STEMI in our emergency department. Coronary angiography was performed using standard techniques. PPCI was performed via the radial artery as the first choice. The patients were immediately given heparin (80–100 U/kg) when the culprit vessel was defined and the intervention decision was made. All PPCIs were performed for the culprit vessel only and according to the lesion anatomy and electrocardiogram, including balloon dilation and stent implantation. Thrombus aspiration and tirofiban infusion were applied according to the operator’s choice. The treatment was considered successful when the coronary artery stenosis was < 50% and the antegrade blood flow of the infarct-related artery achieved a TIMI flow grade of 2 or 3 after PPCI was performed.

### Clinical and laboratory data

We also collected each patient’s medical history, family history, and laboratory dates. Their previous history included hypertension, diabetes mellitus, smoking history, congestive heart failure, stroke, transient ischemic attack, a history of MI, peripheral arterial disease, and complex aortic plaques. Hypertension was defined as a history of hypertension and the use of antihypertensive drugs or the repeated measurement of systolic blood pressure (SBP) ≥140 mmHg and diastolic blood pressure (DBP) ≥90 mmHg. Diabetes mellitus was defined as a previous diagnosis of diabetes mellitus and the use of insulin or antidiabetic agents or a fasting glucose level of > 126 mg/dL. Smoking history was defined as smoking at least one cigarette every day for more than 1 year without cessation. Congestive heart failure was defined as a previous diagnosis of heart failure. Each patient’s family history of coronary heart disease was determined as follows: more than one first-degree relative with heart disease aged ≤55 years for men and ≤ 65 years for women.

The laboratory data included hyperlipidemia (a total cholesterol [TC] level of > 200 mg/dL or low-density lipoprotein cholesterol [LDL-C] level of > 160 mg/dL), hemoglobin (men 130–172 g/L, women 110–150 g/L), creatinine (men 59–104 μmol/L, women 45–84 μmol/L), eGFR,(90–120 ml/min/1.73m^2^), glycosylated hemoglobin (4.8–6.0%), cardiac troponin I [cTnI] (0.01–0.23 ng/L), creatine kinase-myocardial band (CK-MB) (2.0–7.2 ng/L), NT-pro brain natriuretic peptide (NT-pro BNP) (300–450 pg/mL), and left ventricular ejection fraction (LVEF) (50–70%).

### Statistical analysis

Normally distributed continuous variables were expressed as mean ± SD, and group differences were evaluated using the independent-samples *t*-test. Categorical variables were defined as frequencies and percentages, and group differences were evaluated using the Chi-square or Fisher exact test, as appropriate.

Univariate and multivariate analyses were performed to identify the predictors of NRP. Particularly, indicators that may have affected NRP were entered into the univariate logistic regression analyses. However, individual components of the CHA_2_DS_2_-VASc-HSF score and the CHADS_2_ and CHA_2_DS_2_-VASc scores were not entered into the multivariate analysis to avoid multicollinearity. The predictive power of the individual components of the CHA_2_DS_2_-VASc-HSF score was also evaluated using multivariate logistic regression. The results of the regression analyses were presented as odds ratios (ORs) with a 95% confidence interval (CI).

Finally, the sensitivity and specificity of the CHADS_2_, CHA_2_DS_2_-VASc, and CHA_2_DS_2_-VASc-HSF scores in predicting NRP were evaluated at various cut-off values using receiver operating characteristic (ROC) curves. DeLong’s test was used to compare the areas under the curve (AUCs) for the three scores.

A two-sided *p*-value < 0.05 was considered statistically significant. The statistical analyses were performed using SPSS 22.0 software (SPSS Inc., Chicago, IL, USA), except for the comparisons of the AUCs of the three scores, which were performed using MedCalc Statistical software, version 15.6 (MedCalc Software, Ostend, Belgium).

## Results

A total of 454 patients were analyzed: 80 patients in the no-reflow group and 374 patients in the control group. The incidence of NRP was 17.6%. The patients’ demographic characteristics, clinical features, and laboratory findings are summarized in Table 1. There were no group differences in SBP, DBP, heart rate, TC, high-density lipoprotein cholesterol (HDL-C), LDL-C, NT-pro brain natriuretic peptide, hemoglobin, glycosylated hemoglobin, cardiac troponin I, and pain-to-balloon time among the patients. Compared to those of the normal/slow-flow group, the no-reflow group had lower eGFR and LVEF, higher CK-MB and creatinine, and a prevalence of Killip class > 1.

**Table 1:** Demographic characteristics, clinical features, and laboratory findings

Variables	Normal/slow-flow (*n* = 374)	No-reflow (*n* = 80)	*P* value
Age, years, mean (SD)	59.10(11.61)	63.21(11.64)	0.004*
Female sex, n (%)	98(26.2)	18(22.5)	0.491
Hypertension, n (%)	158(42.2)	44(55.0)	0.037*
Diabetes mellitus, n (%)	79(23.4)	25(31.3)	0.143
History of heart failure, n (%)	3(0.8)	1(1.3)	0.539
History of stroke/TIA, n (%)	30(8.0)	17(21.3)	< 0.001*
Vascular disease, n (%)	58(15.5)	30(37.5)	< 0.001*
Smoking, n (%)	216(57.8)	50(62.5)	0.434
Hyperlipidemia, n (%)	97(25.9)	29(36.3)	0.061
Family history, n (%)	48(12.8)	17(21.3)	0.051
eGFR, ml/min/1.73m^2^, mean (SD)	95.92(23.87)	86.25(23.46)	0.001*
SBP, mmHg, mean (SD)	121.86(18.99)	117.83(18.20)	0.083
DBP, mmHg, mean (SD)	76.51(12.64)	75.54(12.34)	0.530
HR, beats/min, mean (SD)	77.58(13.47)	77.18(14.87)	0.809
TC, mmol/L, mean (SD)	4.66(1.06)	4.76(1.06)	0.417
HDL-C, mmol/L, mean (SD)	1.01(0.27)	1.08(0.26)	0.052
LDL-C, mmol/L, mean (SD)	2.99(0.90)	3.04(0.87)	0.665
Creatinine,μmol/L, mean (SD)	69.95(18.95)	78.35(25.18)	0.006*
hemoglobin, g/L, mean (SD)	141.08(17.28)	141.31(15.20)	0.911
glycosylated hemoglobin, %, mean (SD)	6.30(1.45)	6.59(1.53)	0.109
cTnI, ng/L, mean (SD)	1.26(4.92)	4.46(11.84)	0.02*
CK-MB, ng/L, mean (SD)	44.30(74.92)	80.26(103.50)	0.004*
NT-pro BNP, pg/mL, mean (SD)	694.97(2102.8)	1071.8(2519.7)	0.170
Pain-to-balloon time, h, mean (SD)	5.94(3.42)	6.44(2.67)	0.214
CHADS_2_ score, mean (SD)	1.08(1.04)	1.76(1.20)	< 0.001*
CHA_2_DS_2_-VASc score, mean (SD)	1.61(1.38)	2.60(1.58)	< 0.001*
CHA_2_DS_2_-VASc-HSF score, mean (SD)	3.06(1.24)	4.34(1.26)	< 0.001*
LVEF, %, mean (SD)	56.62(8.02)	52.87(9.52)	0.005*
Killip class, n (%)			
1	304(81.3)	56(70.0)	0.024*
> 1	70(18.7)	24(30.0)	

*Abbreviations*: *eGFR* estimated glomerular filtration rate, *TIA* transient ischemic attack, *SBP* systolic blood pressure, *DBP* diastolic blood pressure, *HR* heart rate, *TC* total cholesterol, *HDL-C* high-density lipoprotein cholesterol, *LDL-C* low density lipoprotein cholesterol, *cTnI* cardiac troponin I, *CK-MB* creatine kinase-myocardial band, *NT-pro BNP* NT-pro brain natriuretic peptide, *LVEF* left ventricular ejection fraction, *SD* standard deviation

* *P* < 0.05

The CHADS_2_, CHA_2_DS_2_-VASc, and CHA_2_DS_2_-VASc-HSF scores were significantly higher in the no-reflow group than in the normal/slow-flow group. Four components of the CHA_2_DS_2_-VASc-HSF score—age, a history of stroke/TIA, hypertension, and vascular disease—were higher in the no-reflow group than in the normal/slow-flow group. Other components of the CHA_2_DS_2_-VASc-HSF score, including female sex, diabetes mellitus, a history of heart failure, smoking, hyperlipidemia, and family history, did not differ between the groups.

The patients’ angiographic characteristics are shown in Table 2. Between the no-reflow and normal/slow-flow groups, there was no difference in the patients’ culprit vessels, the prevalence of an initial TIMI flow grade of 0–1, multi-stent, stent length, stent diameter, the use of tirofiban infusion, the use of thrombus aspiration, and the use of an intra-aortic balloon pump. However, multivessel disease was more common in the no-reflow group than in the normal/slow-flow group, but there was no statistical difference.

**Table 2:** Angiographic features

Variables	Normal/slow-flow (*n* = 374)	No-reflow (*n* = 80)	*P* value
Culprit vessel, n (%)			0.765
LM	3(0.8)	1(1.3)	
LAD	228(61.0)	53(66.3)	
RCA	104(27.8)	18(22.5)	
LCX	39(10.4)	8(10.0)	
Multivessel disease, n (%)	135(36.1)	38(47.5)	0.057
Initial TIMI flow grade, n (%)			0.321
0–1	300(80.2)	68(19.8)	
≥2	74(85.0)	12(15.0)	
Multistent, n (%)	153(40.9)	29(36.3)	0.440
Stent length, mm, mean (SD)	39.62(19.83)	43.69(20.33)	0.098
Stent diameter, mm, mean (SD)	3.23(0.46)	3.20(0.42)	0.582
Tirofiban infusion, n (%)	190(50.8)	49(61.3)	0.089
Thrombus aspiration, n (%)	17(4.5)	7(8.8)	0.127
IABP, n (%)	9(2.4)	5(6.3)	0.071

*Abbreviations*: *LM* Left main, *LAD* Left anterior descending, *LCX* Left circumflex, *RCA* Right coronary artery, *IABP* intra-aortic balloon pump, *MI* myocardial infarction, *TIMI* thrombolysis in myocardial infarction, *SD* standard deviation

The results of the regression analyses for the predictors of NRP are shown in Table 3. From the multivariate regression analysis, CK-MB, Killip class, stent length, multivessel disease, tirofiban infusion, and the CHA_2_DS_2_-VASc-HSF score were independent predictors of NRP. The CHADS_2_ and CHA_2_DS_2_-VASc scores were also independent predictors of NRP after adjusting for CK-MB, age, LVEF, eGFR, stent length, stent diameter, multivessel disease, tirofiban infusion, and Killip class (as shown in Table 4). The results of the regression analyses for the individual components of the CHA_2_DS_2_-VASc-HSF score are shown in Table 5. In the multivariate regression analysis, the male sex, hypertension, age 65 to 74 years, age ≥ 75 years, a history of stroke/TIA, vascular disease, family history, and hyperlipidemia were found to be independently associated with NRP.

**Table 3:** Results of the univariate and multivariate regression analyses for the predictors of the no-reflow phenomenon

Variables	Unadjusted OR(95% CI)	*P* value	Adjusted OR(95% CI)	*P* value
LVEF	0.95(0.92–0.98)	0.002	0.98(0.94–1.03)	0.428
Age	1.03(1.01–1.06)	0.005	0.991(0.953–1.03)	0.664
CK-MB	1.004(1.002–1.007)	0.001	1.007(1.003–1.012)	0.002
eGFR	0.982(0.971–0.993)	0.001	0.98(0.97–1.003)	0.093
Killip class	1.86(1.08–3.21)	0.025	0.101(0.02–0.54)	0.007
Stent length	1.01(0.998–1.02)	0.10	1.03(1.01–1.05)	0.012
Stent diameter	0.86(0.504–1.47)	0.581	0.87(0.38–2.04)	0.755
Multivessel disease	1.007(0.61–1.67)	0.979	0.014(0.003–0.08)	< 0.001
Tirofiban infusion	1.53(0.94–2.51)	0.091	0.23(0.07–0.73)	0.012
CHA_2_DS_2_-VASc-HSF score	2.21(1.78–2.75)	< 0.001	31.31(12.53–78.26)	< 0.001

*Abbreviations*: *CK-MB* creatine kinase-myocardial band, *eGFR* estimated glomerular filtration rate, *OR* odds ratio, *LVEF* left ventricular ejection fraction, *CI* confidence interval

**Table 4:** Results of the univariate and multivariate regression analyses for the predictive power of the CHADS_2_, CHA_2_DS_2_-VASc, CHA_2_DS_2_-VASc-HSF scores

Variables	Unadjusted OR(95% CI)	*P* value	Adjusted OR(95% CI)	*P* value
CHADS_2_ score	1.698(1.37–2.11)	< 0.001	2.35(1.59–3.46)	< 0.001
CHA_2_DS_2_-VASc score	1.55(1.31–1.82)	< 0.001	1.80(1.35–2.41)	< 0.001
CHA_2_DS_2_-VASc-HSF score	2.21(1.78–2.75)	< 0.001	31.31(12.53–78.26)	< 0.001

*OR* odds ratio, *CI* confidence interval

**Table 5:** Results of the univariate and multivariate regression analyses for the predictive power of the individual components of the CHA_2_DS_2_-VASc-HSF score

Variables	Unadjusted OR(95% CI)	*P* value	Adjusted OR(95% CI)	*P* value
Male sex	0.82(0.46–1.45)	0.491	0.48(0.22–0.99)	0.048
Hypertension	1.67(1.03–2.71)	0.038	1.82(1.04–3.18)	0.035
Age 65–74 years	2.19(1.26–3.82)	0.006	3.33(1.76–6.32)	< 0.001
Age ≥ 75 years	2.86(1.43–5.71)	0.003	5.52(2.37–12.87)	< 0.001
Diabetes mellitus	1.697(0.995–2.895)	0.052	1.73(0.95–3.17)	0.076
History of heart failure	1.57(0.16–15.2)	0.70	2.06(0.18–23.99)	0.563
History of stroke/TIA	1.76(1.27–2.44)	0.001	2.11(1.45–3.07)	< 0.001
Vascular disease	3.27(1.92–5.57)	< 0.001	4.37(2.39–7.99)	< 0.001
Smoking	1.22(0.74–2.004)	0.435	1.77(0.93–3.38)	0.08
Hyperlipidemia	1.62(0.97–2.71)	0.063	2.71(1.499–4.92)	0.001
Family history	1.83(0.99–3.39)	0.054	2.67(1.299–5.50)	0.008

*Abbreviations*: *TIA* transient ischemic attack, *OR* odds ratio, *CI* confidence interval

**Fig. 1:**
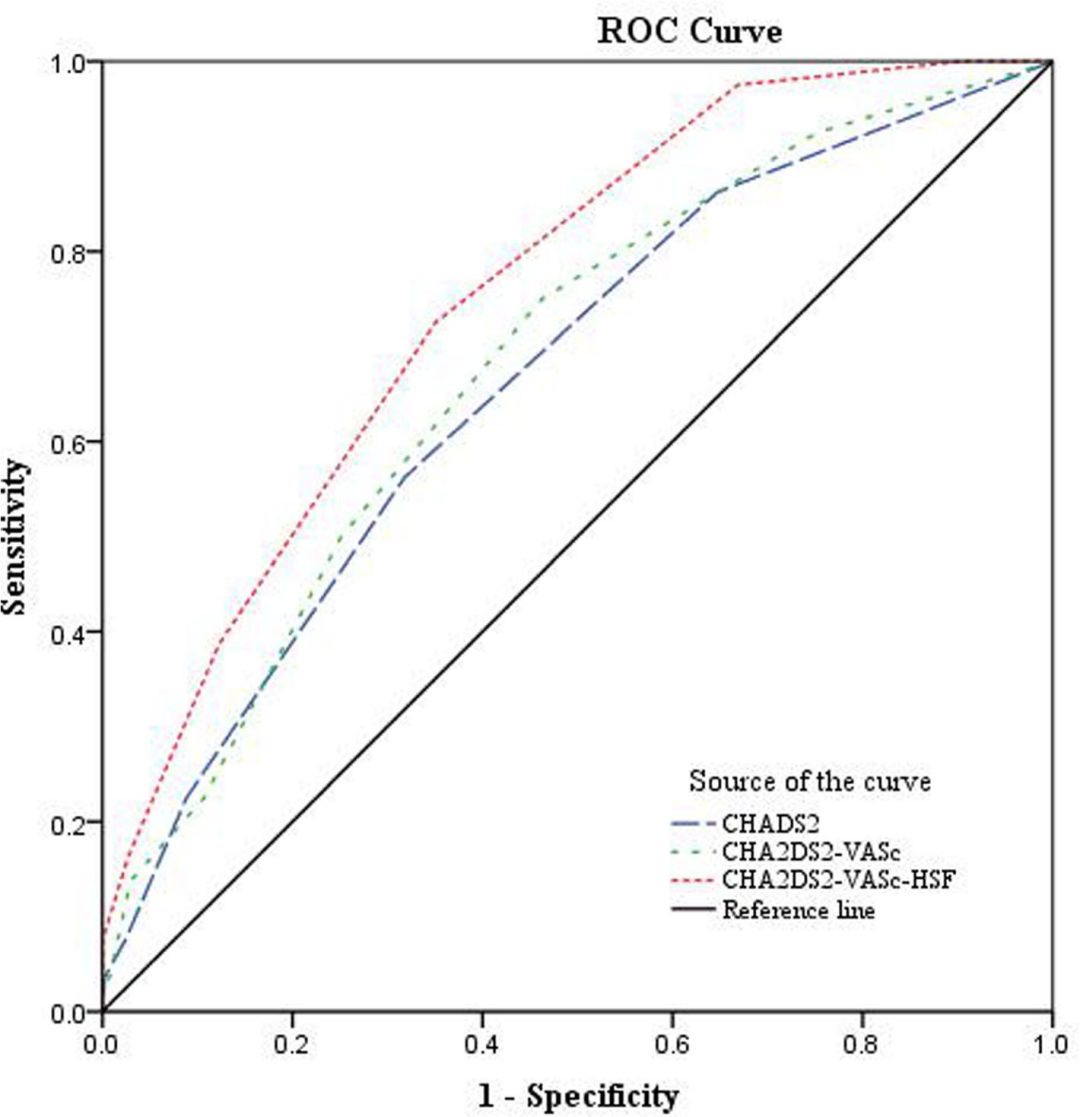
Receiver operating characteristic (ROC) curves of the CHADS_2_, CHA_2_DS_2_-VASc, and CHA_2_DS_2_-VASc-HSF scores in the prediction of the no-reflow phenomenon

The ROC curve analyses revealed that a CHADS_2_ score of ≥2 had a sensitivity of 56.3% and a specificity of 68.2% (AUC: 0.664, 95% CI [0.60–0.73]). A CHA_2_DS_2_-VASc score of ≥2 was also shown to have a sensitivity of 75.0% and a specificity of 53.7% (AUC: 0.682, 95% CI [0.62–0.74]). Finally, a CHA_2_DS_2_-VASc-HSF score of ≥4 had a sensitivity of 72.5% and a specificity of 65.0% (AUC: 0.755, 95% CI [0.70–0.81]), and the score of ≥6 had a specificity of 100% in the prediction of NRP. The ROC curves are shown in Fig. 1. The results of pairwise comparisons in the AUCs are shown in Table 6. The CHA_2_DS_2_-VASc-HSF score was, therefore, found to be the best score for predicting NRP.

**Table 6:** Pairwise comparisons of the areas under the receiver operating characteristics curve

Score	Difference between areas	Standard error	95% Confidence interval	Z Statistic	*P* value
CHADS_2_ vs CHA_2_DS_2_-VASc	0.0173	0.0182	−0.018-0.053	0.95	0.3419
CHADS_2_vsCHA_2_DS_2_-VASc-HSF	0.0907	0.025	0.042–0.140	3.629	0.0003
CHA_2_DS_2_-VASc vs CHA_2_DS_2_-VASc-HSF	0.0734	0.0275	0.0194–0.127	2.666	0.0077

In this study, 239 patients used tirofiban during PPCI. Of these, for the AUC of the no-reflow ROC curve, the CHA_2_DS_2_-VASc-HSF score was 0.763 (95% CI [0.694–0.833]). A CHA_2_DS_2_-VASc-HSF score of ≥4 was the best cut-off value, with a sensitivity of 0.755% and specificity of 0.632%. Conversely, 215 patients did not use tirofiban. A CHA_2_DS_2_-VASc-HSF score of ≥4 yielded a sensitivity and specificity of 0.677 and 0.668%, respectively (AUC: 0.741, 95% CI [0.657–0.826]). In both groups, the CHA_2_DS_2_-VASc-HSF scores are similar predictors of NRP.

## Discussion

This study demonstrates that the CHADS_2_, CHA_2_DS_2_-VASc, and CHA_2_DS_2_-VASc-HSF scores are all independent predictors of NRP after PPCI in patients with STEMI. However, the new CHA_2_DS_2_-VASc-HSF score was found to be a better predictor of NRP than the other two scores, and a score of ≥4 can be used as a cut-off value, with a sensitivity of 72.5% and a specificity of 65.0%. To our knowledge, this is the first study to evaluate the new CHA_2_DS_2_-VASc-HSF score in terms of predicting NRP and comparing the prediction ability of the three scores.

As one of the serious complications of PPCI, NRP can aggravate myocardial ischemia and increase MI size and the incidence of heart failure, in addition to also being an independent predictor of short- and long-term adverse prognoses [4, 5, 21]. The exact mechanism of NRP is still not fully understood, but the mainstream view is that microvascular obstruction, including ischemic injury, reperfusion injury, microvascular dysfunction, and distal microvascular embolization, is the main pathological basis of NRP [4]. Currently, there are few effective therapies for this complication, and no standard treatment has been established to date. Some studies have suggested that a deferring stent has a lower rate of NRP than an immediate stent [22]. Most cardiologists believe that preventing NRP is more effective than treating it. Thus, it is important to determine a simple and effective risk stratification method for NRP that would enable physicians to specify individualized treatment plans before conducting stenting in patients.

The guidelines recommend using the CHA_2_DS_2_-VASc score for stroke risk stratification in patients with atrial fibrillation. However, the components of the CHA_2_DS_2_-VASc score—such as hypertension, diabetes, and age—are well-accepted major risk factors for atherosclerosis. Consistent with this, several previous studies have shown that the CHA_2_DS_2_-VASc score is a predictor of the severity of coronary artery disease [17, 23]. Li et al. [24] also reported that the CHA_2_DS_2_-VASc score is a powerful predictor of major adverse cardio-cerebral vascular events in patients with acute MI, while Wang et al. [10] found that deaths after long-term illnesses, cardiac deaths, and non-fatal strokes are significantly higher in patients with a CHA_2_DS_2_-VASc score of > 2 than in patients with a lower CHA_2_DS_2_-VASc score. Significantly, another previous study concluded that the CHA_2_DS_2_-VASc score is independently associated with NRP and all-cause, in-hospital mortality, [25] which may indicate that the score further predicts patients’ prognoses by predicting NRP. Consistent with this, some of the components of the CHA_2_DS_2_-VASc score are associated with microvascular dysfunction, such as hypertension, diabetes, female sex, and aortic stenosis [26, 27]. Atherosclerosis, heart failure, advanced age, and peripheral vascular disease are additional known risk factors for NRP [28]. Thus, the overlapping of risk factors and shared pathophysiologic pathways may be the reason the CHA_2_DS_2_-VASc score can predict both the prognosis and ischemic events such as NRP in patients. Several other studies have confirmed that the CHA_2_DS_2_-VASc score is an independent predictor of NRP [28–30], and a recent meta-analysis revealed that male sex, a family history of coronary artery disease, and smoking are also associated with NRP [31]. Thus, we sought to evaluate the ability of the new CHA_2_DS_2_-VASc-HSF score to independently predict NRP and compare the prediction ability of the three scores.

Previous studies have identified various predictors of NRP. For example, Bayramoglu et al. [32] and Mazhar et al. [33] found that advanced age, lower-left ventricular ejection fraction, stent length of ≥20 mm, thrombus burden, Killip class ≥3, and longer pain-to-balloon time are independent predictors of NRP. Similarly, anterior infarctions, [34] hypertension, dyslipidemia, a history of smoking, and a history of tobacco use [35] have also been shown to be associated with NRP. In the present study, we demonstrated that Killip class, stent length, and CK-MB are independent predictors of NRP. We also found that multivessel disease, tirofiban infusion, a history of stroke/TIA, family history, and vascular disease are associated with NRP and that the CHA_2_DS_2_-VASc score is an independent predictor of NRP, which is consistent with the findings of previous studies [28–30].

In this study, the multivariate regression analysis did not reveal diabetes mellitus to be a predictor of NRP. A previous study also found that NRP was more likely in patients with hyperglycemia upon hospital admission, while there was no difference in glycosylated hemoglobin or the incidence of diabetes mellitus [36]. Diabetes mellitus is mainly a risk factor of atherosclerosis and is associated with coronary stenosis and microvascular dysfunction. Some studies have also confirmed that diabetes is associated with NRP [37]. In contrast, hyperglycemia may be involved in vascular smooth muscle cell proliferation and migration, oxidative stress, hypercoagulable state, and inflammatory response [38]. Thus, both are associated with the mechanism of NRP, but we believe that it is more likely that admission hyperglycemia is associated with NRP rather than diabetes mellitus. As such, prospective studies with large sample sizes are needed to further clarify this association.

In the present study, male sex was also revealed to be an independent predictor of NRP. A previous study reported that young women with STEMI are more likely than similarly aged men to have reperfusion delays and that the female sex is associated with NRP [39]. This may be due to several factors, such as differences in plaque composition and thrombotic activity between men and women and a higher prevalence of microvascular disease in women than in men. Recently, a meta-analysis of 27 studies found that the male sex is an independent predictor of NRP, which is consistent with the results of the present study. We believe that the male sex may be more closely related to no-reflow; therefore, we changed the female sex in the CHA_2_DS_2_-VASc score to the male sex, which increased the predictive value. This may be because male patients are more likely to smoke and suffer from obesity than female patients.

In the emergency department, STEMI is a common critical illness that requires immediate revascularization. However, if NRP occurs after PPCI, it is dangerous for patients, and treatment is challenging. Thus, a simple and established tool is needed for risk stratification. In the present cohort study, we found that the CHADS_2_ and CHA_2_DS_2_-VASc scores, as well as the new CHA_2_DS_2_-VASc-HSF score, can be independent predictors of NRP since the components of these scores are common risk factors of atherosclerosis, vascular spasm, and microvascular dysfunction, as well as NRP and stroke. Finally, we found that CHA_2_DS_2_-VASc-HSF scores of ≥4 had a maximum predictive value, and the specificity was 100% for CHA_2_DS_2_-VASc-HSF scores of ≥6, which may be helpful in clinic settings.

### Study limitations

The present study has some limitations. It had a single-center, retrospective observational design, and therefore the number of patients analyzed was relatively small. Bias may also exist as the data were mainly based on a review of the previous clinical history of patients in an acute clinical setting. Thus, the calculation of the three scores may have been affected. We did not collect data regarding in-hospital mortality, nor did we conduct patient follow-ups to determine long-term mortality and adverse cardiac events. We had no data regarding pharmacological treatment during or before the procedure, which might influence the achievement of a final TIMI 3 flow. We also had no data regarding the frequency of thrombus aspiration or the use of an intra-aortic balloon pump during PPCI.

## Conclusions

We found that the CHADS_2_, CHA_2_DS_2_-VASc, and CHA_2_DS_2_-VASc-HSF scores are all independent predictors of NRP, but the new CHA_2_DS_2_-VASc-HSF score has higher sensitivity and specificity than the other scores. Thus, the CHA_2_DS_2_-VASc-HSF score may be a simple tool for the prediction of patients at high risk of NRP. Future prospective studies with larger samples are needed to support these results.

## Data Availability

The datasets generated and/or analysed during the current study are not publicly available due to the lack of an online platform but are available from the corresponding author on reasonable request.

## Abbreviations

NRP: No-reflow phenomenon
PPCI: Primary percutaneous coronary intervention
STEMI: ST-segment elevation myocardial infarction
CK-MB: Creatine kinase-myocardial band
HF: Heart failure
TIMI: Thrombolysis in the myocardial infarction
SBP: Systolic blood pressure
DBP: Diastolic blood pressure
TC: Total cholesterol
LDL-C: Low-density lipoprotein cholesterol
NT-pro BNP: NT-pro brain natriuretic peptide
LVEF: Left ventricular ejection fraction
SD: Standard deviation
ORs: Odds ratios
CI: Confidence interval
ROC: Receiver-operating characteristics
AUCs: Areas under the curve
HDL-C: High-density lipoprotein cholesterol
